# Death: Its Modes, Signs and Premonitions

**Published:** 1890-07

**Authors:** F. Bradnack

**Affiliations:** Buffalo, I. N. D.


### DEATH: ITS MODES, SIGNS, AND PREMONITIONS.

By F. BRADNACK, M. D.

(*Concluded from June number.*)

### PREMONITIONS.

With some persons the first symptom of approaching death is the strong presentiment that they are about to die. Ozanam, the mathematician, while in apparent health rejected pupils, from the feeling that he was on the eve of death. Soon after he expired of an apoplexy. Flechier, the divine, had a dream which shadowed out his impending dissolution, and believing it to be a merciful warning from heaven, he sent for a sculptor and ordered his tomb. "Begin your work forthwith," he said; "there is no time to lose." His speedy decease proved that his premonitions were not unfounded. Mozart wrote his immortal *Requiem* under the conviction that it would be for himself. When life was flitting fast, he called for the score, and musing over it, said: "Did I not tell you truly that it was for myself that I composed this death-chant?" Another great artist, in a different department, having a premonition that his possibility of work was near its end, chose a subject emblematical of the coming event. His friends inquired the nature of his next design, and Hogarth replied, *The End of All Things*. "In that case," rejoined one of the number, "there will be an end of the painter." To this jesting rejoinder Hogarth replied, "There will, and therefore the sooner my work is done the better." He began next day, labored at the picture with unusual assiduity, and when he had given it the last touch, seized his palette, broke it in pieces, and said, "I have finished." The print was published in March, under the title of "Finis;" and in October Hogarth was dead.

In explanation of these and similar premonitions, John Hunter said: "We sometimes feel within ourselves that we shall not live, for the living powers become weak, and the nerves communicate the intelligence to the brain." His (Hunter's) own death seemed to be a confirmation of this dictum. He intimated, on leaving home, that if a discussion (which awaited him at the hospital) took an angry turn, it would prove his death. A colleague gave him the lie. The coarse word verified the prophecy, and he expired almost immediately in an adjoining room.

On the approach of death many noticeable traits occur. Some, as the last hour draws nigh, toss the clothes from the chest. Very often have I observed this as a premonitory mortal sign; and so, I presume, have most physicians. The attendants, with a blameworthy assiduity, are usually indefatigable in replacing these clothes, doubtless to the distress of the patient; for they are as often thrust back as replaced. There must be some oppression in the covering, or it would not be so persistently thrown off; though, probably, the efforts are instinctive rather than conscious. Another frequent premonitory sign is *picking at the bedclothes*, or at some imaginary object, the latter constituting, of course, an optical illusion. Falstaff fumbled with the bedclothes, we are told by gossiping Dame Quickly. *Muscæ volitantes* appear frequently to trouble the vision of the dying. The ears, too, are often assailed by imaginary voices, which are inaudible to the bystanders, — this, however, is altogether a different thing from saying that they are non-existent voices. Of course, the sceptic and the atheist will put them down as "delusions," and "imaginations," that cheap refuge of those who deny everything unverifiable by the five senses. Often when the sensibility to outward impressions is lost, and the mind is semi-delirious, the dying dream of their habitual occupations, or revert to scenes in their past lives. Napoleon fought again some battle, and his last words, *tête d'armèe*, were characteristic. The celebrated Dr. Armstrong departed delivering medical precepts. Lord Tenterden, who passed from the judicial bench straight to his death-bed, fancied himself still presiding at a trial; and died exclaiming: "Gentlemen of the jury, you will now consider of your verdict." A great French jurist, Malherbe, whose grammatical pedantry haunted him through life, split, as one might say, philological hairs in his last gasp. When near his end, he rallied his last iota of strength to correct a bystander for an inelegance of diction. Being rebuked by his confessor for this levity, he declared that he could not help it; for, he felt bound *défendre jusqu' á la mort la pureté de la langue Française.*

Augustus Cesar, when about to expire, after sending for a mirror and arranging his hair, asked jestingly whether he was not a good comedian. Indeed the different manners in which different persons approach death are both curious and interesting. Madame de Pompadour met it apparently with ostentatious indifference. She put on a silk dress, painted her face (like Pope's *Narcissa*), had her hair coiffed; and, as her confessor was about to leave, stopped him exclaiming: *Attendez un instant, M. le Curè; nous nous en irons ensemble!* Nothing can much surpass this, unless it be the cynicism of her royal lover (now grown cold), who on seeing her funeral procession, coolly remarked: *Madame la Marquise aura aujourd'hui un maurais temps pour son voyage.* Very different from the last words of the brilliant Frenchwoman were (if tradition be correct) those of the English queen, Elizabeth: "All my possessions for a moment of time!"

Wit often flashes, meteor-like, in the closing moments. A Frenchman, in his last illness, being wearied by the importunities of a priest, silenced him with the promise: *Vous serez payè, mais laissez-moi en repose.* Charles Lamb said he hoped that his own last breath would be inhaled through a pipe, and exhaled in a pun. The "ruling passion strong in death" is well illustrated in the last words of Napoleon (*tetè d'armée*); of Haller (*the artery ceases to beat*), and of Haydn (*God preserve the Emperor*), doubtless thinking of his own sublime hymn to those words, which afterward became the national hymn of Austria. Gassendi, who was more philosophical than religious, exclaimed when near his end, "I know neither who placed me in the world, nor why I was placed in it, nor why I am taken from it." (Possibly, by this time, he has found out.) Very different were the last words of the erudite, the philosophic, and the truly religious Swedenborg. Having inquired of a female attendant what hour it was, he calmly rejoined: "It is well; I thank you; God bless you," and expired in the same sure and certain hope of immortality in which he had lived and labored for more than eighty years. Rameau, the French composer, whose well-trained ear was extremely sensitive to inharmonious sounds, was, on his death-bed, visited by a priest who chanted, all out of tune, the mass in the dying man's ears. Rameau roused up and said: "*Que diable ferez–vous me chanter la? Vous avez la voix fausse!*" Lord Palmerston, in his last illness, when the physician felt called on to mention the possibility of death, replied: "Die! my dear doctor, that's the last thing I shall do."

Mozart (as aforesaid) had a very distinct premonition of his death. On the night of 5th December, 1791, when his wife's sister arrived, he said to her: "O, my dear Sophie, it is well that you are come; you must stay to-night, and you must see me die. I have the taste of death on my tongue; I smell the grave."

Curious forebodings and foreshadowings of impending death are sometimes experienced. For instance, the learned Conrad Gesner one night dreamt that he was bitten on the left side by a venomous serpent. In a short time a severe carbuncle appeared on the identical spot, and death ensued in five days.

In many instances the mind is capable of pursuing a beaten track without attending to its own operations, and the least impulse will set it going when all its other powers have fled. For instance, the mathematician, DeLagny, was asked the square of twelve when he was unable to recognize the friends around his bed, and mechanically answered, "One hundred and forty-four." The idea of Dr. Adam that it was growing dark, evidently arose from the fading away of the power of sight, as the darkness of death came down upon him. Doubtless Goethe experienced the same increasing obscuration, for his last words were, "Let the light enter!"

Fatal maladies appear sometimes to make a sudden stop; the patient seems as if about to recover, and the friends who considered him moribund, rejoice that he is once more himself. But experienced physicians know that this is the "lightening before death," and know also that it is a fallacy of appearance. The medical attendant of Charleval, a French poet, called out exultingly to a brother of the faculty, who entered the room, "Come and see; the fever is going!" After a moment's examination the other, more experienced, replied, "No, it is the patient."

Of the many roads which lead to death some are easier to travel than others; and if a choice could be had, doubtless there would be much difference of opinion as to which was the easier. Both Cesar and Pliny desired an instantaneous death, and Augustus had a similar wish. Montaigne also held to Cesar's view, and, to use his own quaint metaphor, thought the pill was swallowed best without chewing. During the plague of Syracuse many of the Romans attacked the posts of the enemy in order that they might die by the sword rather than by the pestilence. Gustavus Adolphus used to say that no man was happier than he who died in the exercise of his vocation. He himself died on the battlefield of Lutzen. The gallant Nelson also wished the roar of cannon to sound his parting knell. He, too, had his desire. When Cavendish, the great chemist, perceived that his end drew near he ordered his attendant to retire and not to return until a certain hour. The servant came back to find his master dead.

Premonitions are of many kinds, and are manifested in various ways. Dr. Fidge, a physician of the old school (who, in early days, had accompanied William IV. when he was a midshipman, as medical attendant,) possessed a favorite boat. On his retirement from Portsmouth dockyard, where he held an appointment, he had the boat converted into a coffin. This coffin he kept under his bed for many years. The circumstances of his death were somewhat remarkable. He had, with his solicitor's aid, after a paroxysm of pain, just added a codicil to his will. The lawyer then asking him how he felt, he replied, "As easy as an old shoe;" and looking toward the nurse in attendance said: "Just pull my legs straight and place me as a dead man, it will save you trouble shortly,"—words which he had scarcely uttered before he calmly died.

Again, some premonitions appear to take the form of coincidences, and appear quite inexplicable. For instance, Shilling records that a young man in Padua dreamt one night that he was bitten by one of the marble lions which stand before the church of St. Justina. Passing by the place on the following day with some companions, he recalled the dream, and, putting his hand into the mouth of one of the lions, he said, defiantly, "Look at the fierce lion that bit me last night!" But, at the same moment he uttered a piercing cry, and drew back his hand in great terror. A *scorpion*, hid in the lion's mouth, had stung him, and the poor youth died of the venom.

That profound thinker, Kant, admitted the probability that ere long the process will be discovered by which the human soul, even in this life, is closely connected with the immaterial inmates of the world of spirits. Religion had already asserted that it was so connected. Lord Byron, who was sceptical enough in many respects, nevertheless says, "I am a great believer in presentiments. Socrates's demon was no fiction. Monk Lewis had his monitor, and Bonaparte many warnings," to which admission might be added the old German saw, which says:

> "Who neither believes in heaven nor hell
> The devil heartily wishes well."

I am disposed, from facts which have come to my knowledge, to regard the howling of a dog as sometimes a premonition of death to some member of the family. Both in England and Germany dogs are reported in innumerable instances to have set up a most painful howling before the approaching death of inmates of a house where they were kept; and they are thought to see supernatural beings, when they are seen to cower down of a sudden and to press close to the feet of their masters, trembling and looking up piteously, as if for help. Popular belief then says: "All is not right with the dog," or "he sees more than men can see." Samuel Wesley tells us how a dog, specially brought up for the purpose of frightening away the evil-disposed men (who were at first suspected of causing the nightly disturbances at the parsonage), barked but once the first night, and after that exhibited, on the occurrence of those noises, quite as much terror as the children.

In some cases premonitions occur but a short time prior to dissolution. The King, George IV., was, by his physicians, entertained with false hopes of recovery in his last illness. "Wally," said the King, "what is this? *This is death*, my boy. They have deceived me." These words, which were his last, were addressed to his page, Sir Waltham Waller, who was assisting the King to a seat when the final qualm came.

Lord Thurlow received a sudden premonition, and his last words were, to say the least, singular. After having lain quietly for some time, he suddenly raised himself in bed, and exclaimed: "I'm shot if I don't believe I'm dying!" And his surmise was correct. He was.

It is well known that often dying persons not only hear voices which are inaudible to their friends and attendants, but also see, or appear to see, persons or forms which are invisible to others. I have myself been a witness to this experience on the part of moribund patients. Infidels, atheists, and sceptics, who are filled with doubts, sneers, floutings, and Abderian laughter, (to whom might often be applied the words of Plautus: *stulti stolidi fatui fungi bardi blenni buccones,*) these men put all such death-bed phenomena down to what they call "delusion and imagination," those easy refuges of such as can neither deny facts nor explain them. Charles Lamb's parody on Shakspere's couplet not only serves them right but fits them well:

"Full fathom five the *atheist* lies,
Of his bones are hell-dice made."

There is certainly nothing about them that savors of heaven. For my own part I do not hesitate to aver that I am not of their way of thinking. To my mind it would be as impossible, as it would be unphilosophical, to suppose that the innumerable instances on record of such sounds and sights are mere imaginings or delusions, for many of them have been experienced by persons in no way given to imagination or to being imposed on by delusions. They have even been experienced by young children. In short, there appears to be no doubt that, as the bodily senses grow dull toward death, so do the faculties of the spirit — the real man — increase in activity and perceptive power. In a word, the spiritual eyes and ears are opened to see and to hear what, at other times, is invisible and inaudible. Again, Shakspere, who saw clearly what other men see dimly, confirms this theory, where he says, speaking of actual although inaudible celestial music, — "For, while this muddy vesture of decay doth closely hem us in, we cannot hear it," — the "muddy vesture" of course referring to the corporeal body. Doubtless all this can be denied or disputed, but, for the matter of that, so can almost anything else. To deny a phenomenon simply because *we* cannot explain it is as contrary to philosophy as it is to common sense; for it must needs be that in an infinite universe there are many things incomprehensible to merely finite intelligences. To deny facts simply because *we* cannot explain them, would be as senseless as to deny the existence of the Atlantic ocean because we cannot bale it out with a pint cup.

Said that great and good man, John Wesley, when speaking of a reported case of supernatural visitation, which had been narrated to him: "It is true there are several of the circumstances I do not comprehend, but this is with me a very slender objection, for *what is it which I do comprehend*, even of the things which I see daily? Truly not the smallest grain of sand or spire of grass. I know not how one grows or how the particles of the other adhere together. What pretense have I, then, to deny well-attested facts because I cannot comprehend them?" Surely it would serve a useful purpose if these wise words could be written in gold and set up for the benefit of the various infidels, deists, sceptics, atheists and agnostics, which now-a-days crop up as plentiful as blackberries, and flourish like weeds growing by a cesspool. Possibly, also, the word sof a greater man than even John Wesley might sometimes apply to some of them, puffed up with vanity and self-conceit as many of them are, "Seeking to be wise they became fools." But, lest the erudite and pious Wesley should not be scientific enough for these atheistical and agnostical gentry, who usually affect a lofty, ultra-scientific scorn of any opinions not emanating from their own clique, let them perpend the words of one of the greatest scientists, as well as philosophers, the famous Laplace: "We are so far from knowing," says this great man, "all the agents of nature and their various modes of action, that it would not be philosophical to deny any phenomena merely because in the actual state of our knowledge they are inexplicable. This only we ought to do,—in proportion to the difficulty there seems to be in admitting them, should be the scrupulous attention we bestow on their examination."

Let the infidels, atheists, agnostics, sceptics, positivists (*et id malum genus omne*) memorize this dictum. To digest it cannot fail to benefit them.

The Roman poets believed that every man possessed a three-fold soul, which, after the dissolution of the body, resolved itself into (1) the *manes*, (2) the *anima* or *spiritus*, (3) the *umbra*, to each of which a different place was assigned. The *manes* descended into the infernal regions, to inhabit either Tartarus or Elysium; the *anima* ascended to the skies, while the *umbra* hovered around the tomb, as if unwilling to quit its connection with the body, of which it was the wraith or shadow. The "spirit" is the *nous* of Plato; the immortal, immaterial, and purely divine principle in man, the crown of the human triad, referred to as the "body, soul and spirit" by St. Paul. The "soul" is the *psyche* or the *nepheth* of the Bible; the vital principle, or the breath of life, which every animal shares with man. Prof. Schele de Vere truly says: "There prevails among all men, at all ages, a carefully repressed but almost irresistible belief in supernatural occurrences, in the close proximity of the spirit world. This belief is neither to be treated with ridicule, nor to be objected to as unchristian, since it is an abiding witness that men entertain an ineradicable conviction of the immortality of the soul."

Who, we may ask, can dispute this? It is as much a hard scientific fact as that two parts of hydrogen and one part of oxygen go to the production of a molecule of water. In this connection a recent able writer (F. C. Cobbe) says: "I have heard numberless instances of dying persons showing unmistakably by their gestures, and sometimes by their words, that they see in the moment of dissolution what could not be seen by those around them." This writer then gives the following case, witnessed by herself. The sick man was the relator's own brother. "He was," says she, "an elderly man, dying of a painful disease, but one which never for a moment obscured his faculties. Although it was known to be incurable, he had been told that he might live some months, when, somewhat suddenly, the summons came on a dark January morning. It had been seen in the course of the night that he had been sinking, but for some time he had been perfectly motionless, apparently in a state of stupor, his eyes closed and his breathing scarcely perceptible. As the tardy dawn of the Winter morning revealed the rigid features of the countenance, from which life and intelligence seemed to have quite departed, those who watched him felt uncertain whether he still lived; but suddenly, while they bent over him to ascertain the truth, he opened his eyes wide and gazed eagerly, with such an unmistakable expression of wonder and joy that a thrill of awe passed through all who witnessed it. His whole face grew bright with a strange gladness, while the eloquent eyes seemed literally to shine, as if reflecting some light on which they gazed. He remained in this attitude of delightful surprise for some minutes; then in a moment the eyelids fell, his head drooped forward, and, with one long breath, the spirit departed."

It would appear, in some rare instances, that after the immortal spirit has been loosed from its "muddy vesture of decay," it has returned to reanimate it for a brief moment, before taking its final departure. There is an account of an old man who had left some generous gifts to his orphaned nieces in his will, which document, just before his death, he had confided to his rich son, with injunctions to carry out his wishes. But he had not been dead more than a few hours, before the son, finding himself alone with the corpse, tore the will and burned it. The sight of this impious deed apparently recalled the hovering spirit (or at least the *umbra* of the Romans), and the old man, rising from his couch of death, uttered a fierce malediction on the horror-stricken wretch, and then fell back again and yielded up his spirit, this time forever.

Though medicine has many resources which those unacquainted with its powers are incapable of estimating, still its functions are limited, and, although the wise physician can often cure the sick he never pretends to cure the dead; for he knows that he possesses none of that *promethean unguent*, such as Medea gave to Jason, whereby the recipient was rendered invulnerable to the darts of death. We possess as yet no *elixir vitæ*. Alas, no! Death is the great *crux medicorum;* for, as the proverb truly says: To prescribe physic for the dead and advice for the old is the same thing. In strict truth, the physician who lays the flattering unction to his soul that *he* cures sick people is most egregiously mistaken, for, certes, it is God who cures the patient, while the doctor puts the fee in his pocket. We give ourselves great airs of wisdom with our diagnoses, and prognoses, and scientific therapeutics, but did not the *vis medicatrix naturæ* come to our assistance, the wisest physician would, so far as the patient is concerned, be but little better than the most illiterate quack.

In conclusion, when we finally reach (as we each and all shall reach) that *ripa irremeabilis undæ*, of which Virgil spake, both medicine and physicians will be in vain. To the same oblivion we are all hastening, the great and the small, the rich and the poor, the wise and the foolish—to the same last resting-place. To such as do not relish the thought of a bed so narrow, and a lodging so humble, the German adage may possibly afford some slight measure of consolation. *He*, says this medieval saw, *who lies in the grave is well lodged.* At least his body will be well lodged. How his immortal soul may fare is another matter which lies outside our present province.

"Here lies one whose name is writ in water," wrote Keats for his own epitaph, and surely it might well serve for the universal epitaph of all mankind. Many people complain of the uncertainties of life, and, of a truth the life of man is a winter's day and a winter's way. But, if life may fairly be reproached with uncertainty, death cannot be so reproached; for, of all human events, it is the most certain. Once launched upon its lethean stream, the king and the slave are fairly embarked on the same voyage to oblivion. Some will arrive there sooner than others, but for each one it is only a question of time. "*Veneatapadino Ragium!* how few," says Jeremy Taylor, "ever heard of him; and yet he was the great King of Narsinga!"

—and, truly, the preacher might have added that, for all we know to the contrary, his royal ashes may to-day be stopping a leak in a beer barrel. "Now, get you to my lady's chamber, and tell her let her paint an inch thick, to this favor she must come; make her laugh at that."

Yet, due to a kind Providence, to many sufferers death comes as by no means an unwelcome messenger, for "Death is the liberator of him whom freedom cannot release; the physician of him whom medicine cannot cure, and the comforter of him whom time cannot console."

The last hour will surely come. *God's club*, says the Russian proverb, *makes no noise, but when it strikes there is no cure for the blow.* The majority of mankind, however, regard death as their worst enemy, and shrink from the time when they shall become *pabulum Acherontis.* Even Charles Lamb, who was very far from being worldly-minded, dreaded the uncertainty regarding our future state. He seemed to feel a palpable horror at the thought of going to an unknown, an undiscovered country. "God help me," says he, "when I come to put off these snug relations and get abroad into the world to come! I shall be like the crow in the sand, as Wordsworth has it."

Finally, it may in all truth be said that dying is as natural as living; and, to those who find this world an unsatisfactory abiding place — and their name is legion — and who do not share in the fears of death entertained by the many, may be commended the consolatory words of the Persian poet. "If," says he, "this world were our eternal abiding place, we might complain that it makes our bed too hard. But it is only our night-quarters on a journey, and who can, under such circumstances, expect home comforts?" And to those who, so far from dreading death, are anxiously awaiting it as a remedy for ills which are immedicable in this life, may be quoted the consolatory and patience-inspiring line from Pliny: *Longissimus dies cito conditur.*

However, whether we dread it or look forward to it with joy, the day will inevitably come for each of us when it must be said, as the old Romans used to say:

APIO OPUS EST.

BUFFALO, I. N. D., Xmas, 1889.

---

THE health officer of Chicago has refused to accept "heart failure" as a cause of death. It is said that one hundred and fifty death certificates so signed have been returned, with a request for information as to the true cause of death.—*Times and Register.*

